# Texting while driving: the development and validation of the distracted driving survey and risk score among young adults

**DOI:** 10.1186/s40621-016-0073-8

**Published:** 2016-03-01

**Authors:** Regan W. Bergmark, Emily Gliklich, Rong Guo, Richard E. Gliklich

**Affiliations:** 1 Clinical Outcomes Research Unit, Massachusetts Eye and Ear Infirmary, 243 Charles Street, Boston, MA 02114 USA; 2 Department of Otolaryngology, Massachusetts Eye and Ear Infirmary, 243 Charles Street, Boston, MA 02114 USA; 3 Harvard Medical School, Boston, MA USA

## Abstract

**Background:**

Texting while driving and other cell-phone reading and writing activities are high-risk activities associated with motor vehicle collisions and mortality. This paper describes the development and preliminary evaluation of the Distracted Driving Survey (DDS) and score.

**Methods:**

Survey questions were developed by a research team using semi-structured interviews, pilot-tested, and evaluated in young drivers for validity and reliability. Questions focused on texting while driving and use of email, social media, and maps on cellular phones with specific questions about the driving speeds at which these activities are performed.

**Results:**

In 228 drivers 18–24 years old, the DDS showed excellent internal consistency (Cronbach’s alpha = 0.93) and correlations with reported 12-month crash rates. The score is reported on a 0–44 scale with 44 being highest risk behaviors. For every 1 unit increase of the DDS score, the odds of reporting a car crash increases 7 %. The survey can be completed in two minutes, or less than five minutes if demographic and background information is included. Text messaging was common; 59.2 and 71.5 % of respondents said they wrote and read text messages, respectively, while driving in the last 30 days.

**Conclusion:**

The DDS is an 11-item scale that measures cell phone-related distracted driving risk and includes reading/viewing and writing subscores. The scale demonstrated strong validity and reliability in drivers age 24 and younger. The DDS may be useful for measuring rates of cell-phone related distracted driving and for evaluating public health interventions focused on reducing such behaviors.

## Background

Texting and other cell phone use while driving has emerged as a major contribution to teenage and young adult injury and death in motor vehicle collisions over the past several years (Bingham 2014; Wilson and Stimpson 2010). Young adults have been found to have higher rates of texting and driving than older drivers (Braitman and McCartt 2010; Hoff et al. 2013). Motor vehicle collisions are the top cause of death for teens, responsible for 35 % of all deaths of teens 12–19 years old, with high rates of distraction contributing significantly to this percentage (Minino 2010). In 2012, more than 3300 people were killed and 421,000 injured in distraction-related crashes in the US, with the worst levels of distraction in the youngest drivers (US Department of Transportation National Highway Traffic Safety Administration 2014).

While distracted driving includes any activity that takes eyes or attention away from driving, there has been particular and intense interest on texting and other smartphone-associated distraction as smartphones have become widely available over the past ten years. Multiple studies have examined driving performance while texting or completing other secondary tasks (Yannis et al. 2014; Owens et al. 2011; Olson et al. 2009; Narad et al. 2013; McKeever et al. 2013; Drews et al. 2009; Hickman and Hanowski 2012; Leung et al. 2012; Long et al. 2012). Uniformly, distraction from cell phone use, including texting, dialing or other behaviors, is associated with poorer driving performance (Yannis et al. 2014; McKeever et al. 2013; Bendak 2014; Hosking et al. 2009; Irwin et al. 2014; Mouloua et al. 2012; Rudin-Brown et al. 2013; Stavrinos et al. 2013). A 2014 meta-analysis of experimental studies found profound effects of texting while driving with poor responsiveness and vehicle control, and higher numbers of crashes (Caird et al. 2014). A rigorous case–control study found that among novice drivers, sending and receiving texts was associated with significantly increased risk of a crash or near-crash (O.R. 3.9) (Klauer et al. 2014). In commercial vehicles, texting on a cell phone was associated with a much higher risk of a crash or other safety-critical event, such as near-collision or unintentional lane deviation (OR 23.2) (Olson et al. 2009). Motor vehicle crash-related death and injury have also been strongly associated with texting (Pakula et al. 2013; Issar et al. 2013).

Although the dangers of texting and driving are well-established, a focused brief survey on driver-reported texting behavior does not yet exist. Multiple national surveys which include texting while driving as part of a more extensive survey on distracted driving or youth health have found that young drivers have high rates of texting while driving, often in spite of high levels of perceived risk (Hoff et al. 2013; Buchanan et al. 2013; Cazzulino et al. 2014; O’Brien et al. 2010; Atchley et al. 2011; Harrison 2011; Nelson et al. 2009). The surveys confirm that young adults are at high risk for distracted driving; in one, 81 % of 348 college students stated that they would respond to an incoming text while driving, and 92 % read texts while driving (Atchley et al. 2011). Among several large survey based studies, the National Highway Traffic Safety Administration reported from a 2012 survey that nearly half (49 %) of 21–24 year old drivers had ever sent a text message or email while driving (Tison et al. 2011-12), and even more alarming, the Centers for Disease Control and Prevention (CDC)’s National Youth Risk Behavior Survey found that nearly as many high school students who drove reported texting in just the past 30 days (41.4 %) (Kann et al. 2014). The problem is not confined to novice drivers. Among US adults ages 18 to 64 years 31 % report reading or sending text messages or emails while driving in prior last 30 days (Centers for Disease Control and Prevention (CDC) 2013).

Given the magnitude of the problem, a very brief questionnaire focused on texting and driving for evaluation of public health measures such as anti-texting while driving laws, cell phone applications and public health campaigns would be useful. The use of self-reported validated surveys is an increasingly common approach to understanding health issues as well as their response to intervention (Guyatt et al. 1993; Tarlov et al. 1989; Stewart and Ware 1992). Current surveys are driving-specific but lengthy and potentially prohibitive for widespread dissemination (Tison et al. 2011-12, McNally and Bradley 2014; Scott-Parker et al. 2012; Scott-Parker and Proffitt 2015), do not include texting as a survey domain within the realm of distraction (Martinussen, et al, 2013), are general health surveys without sufficient information on texting and driving (Kann et al. 2014), or have not been designed or validated to reliably measure and evaluate individual crash risk (Kann et al. 2014). For example, a new survey of reckless driving behavior includes information on multiple driving-related domains of behavior, but administration takes 35 min and the survey does not focus on cell phones (McNally and Bradley 2014). Another survey of distraction in youth is similarly comprehensive without a focus on phone use (Scott-Parker et al. 2012; Scott-Parker and Proffitt 2015). The goal of shorter surveys for evaluation of distracted driving has been well documented and development of the mini Driver Behavior Questionnaire (Mini-DBQ) is an example, although it does not address cell phone related distracted driving (Martinussen et al. 2013). However, many interventions target cell phone use specifically rather than distraction broadly. In addition, most surveys do not delve into the specific timing of texting while driving that allows a more precise estimate of the behavior’s prevalence.

The purpose of this study was to develop a reliable self-reported survey for assessing levels of cell phone related distracted driving associated with viewing and typing activities and to validate it in a higher risk population of drivers age 24 years or younger.

## Methods

### Study design and oversight

A literature review and open-ended interviews with experienced and novice drivers were performed to identify the most common domains for item development as well as any existing survey items with validation metrics. The literature review was performed with reviewing terms including “Text*” and “Driv*” reviewing for any studies that included driver-reported outcomes. Initial items were piloted with open-ended responses. Ten novice (18–25 years old) and experienced (30 years old or older with at least 10 years of driving experience) drivers underwent semi-structured interviews about cell phone use while driving to further generate potential survey domains. Text messaging through various applications, map/GPS use, email and social media were prominent themes. “Texting while driving” was interpreted very differently by various participants; some people stated that texting at stop lights or at slow speeds, or reading texts, did not really constitute texting and driving. This finding suggested that a questions that simply asks “do you text and drive?” may be missing a significant proportion of this distracted behavior.

Based on the identified themes, we developed a series of Likert scale and multiple-option items reflecting the most common reading and typing tasks reported on a cell phone (Table 1). The format of many of our questions was modeled on the Centers for Disease Control and Prevention National Youth Risk Behavior Survey and after a thorough review of the other surveys described above. The assessed activities included reading or viewing text messages, emails, map directions, internet sites and social messaging boards and typing or writing activities through these same applications. The piloting process revealed that in addition to questions addressing frequency of the activity over the previous 30 days while driving (e.g. every time, most of the time, etc.), it was important to also assess when the activities were performed with respect to vehicular motion or speed (any speed, low speeds, stop and go traffic, etc.) to allow for further risk stratification. Additional items assessed driver attitudes with respect to their perceived level of risk associated with performing these activities. The questionnaire was pre-tested with 30 drivers 18–24 years old and went through multiple iterations. In addition to questions on cell phone reading and writing activities, the questionnaire included demographic information, self-reported “accidents” within the past 12 months of any cause, and potentially high-risk activities such as driving under the influence of alcohol or other substances. Given the colloquial use of the phrase “car accident,” we used the term “car accident” in our survey, but in the results section refer to this number as the crash rate. The question included in the final survey to elicit crash data was, “In the last 12 months, have many car accidents have you been in with you as the driver? (Answers 1, 2, 3, 4, 5 or more).” Based on feedback from the pilot testing, twenty-nine items were selected for testing in the initial questionnaire.

**Table 1:**
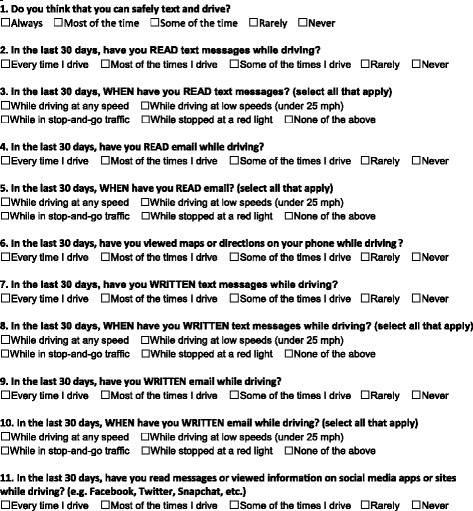
Distracted driving survey

The questionnaire was set up as a web-based survey using standard, HIPAA compliant software. Participants provided informed consent and received a nominal incentive for participating. The study was approved by the Massachusetts Eye and Ear Institutional Review Board.

### Participants

Three pools of participants 18–24 years old who had driven in the prior 30 days were recruited: (1) greater Boston metropolitan area were recruited from educational or recreational centers in the greater Boston area with flyers, enrolled through a generic link, and completed a second survey at 14 days for test-retest reliability, after which several questions were eliminated yielding and 11-item questionnaire (2) A panel was used through the software program to recruit participants from two geographic locations, (a) Eastern and (b) Western United States for a larger geographical distribution for further validation. These participants completed the survey a single time.

### Item selection: reliability and validity

With the goal of creating a brief and targeted survey, items were selected for inclusion in the total score based on multiple reliability and reliability measures (Table 1). Item response distribution was examined prior to analysis. Items with low test-retest reliability in the Boston sample defined as a Spearman correlation of less than 0.4 or a Kappa coefficient below 0.3 were eliminated. Internal consistency was measured with Cronbach’s alpha, examining Cronbach’s alpha for each item and the DDS coefficient with each variable deleted, with any questions with a Cronbach’s alpha under 0.8 eliminated. In addition to face validity, the survey was assessed for criterion-related validity by use of concurrent validity against hypothesized correlates to other assessed variables. We hypothesized a significant correlation to self-reported crashes in the prior 12 months. We additionally postulated that writing related activities would be higher risk than reading or viewing activities alone. Conversely, we hypothesized non-significant correlations with other items (e.g. falling asleep while driving).

Items not focused on cell phone writing and reading behaviors or crash rate also were eliminated from the final survey to allow for brevity. The final survey was then tested in two cohorts of young drivers to confirm internal consistency, time required for survey completion and correlation with crash rate.

### Statistical analysis

All data analysis was performed using SAS V9.4 (SAS Institute Inc., Cary, NC). Standard descriptive statistics were reported, mean (SD) for numerical variables, median (min – max) for Likert scale variables and frequency count (%) for categorical variables. The statistical underpinnings of patient-reported outcomes measures and survey design are well established; the reader may reference Fleiss’s Design and Analysis of Clinical Experiments for a detailed discussion of the methods chosen for this study (Fleiss 1999).”

An algorithm was created to generate a total Distracted Driving Survey (DDS) score based on the final items selected for the questionnaire where zero represents the lowest possible score. The response for each of the questions included was given a value 1–5 with 1 being the lowest risk answer (ie, no texting and driving) and 5 being the highest risk. For a given subject, the scores for the questions were then summed and reduced by the number of questions such that the lowest score was zero. The final survey, consisting of 11 questions, therefore had a range of possible scores ranging from 0 to 44, with 44 being the highest risk. In addition, two subscores for reading only (DDS-Reading) and writing only (DDS-Writing) related questions were created for further risk stratification based on evidence that writing texts is even more dangerous than reading texts alone (Caird et al. 2014). Wilcoxon tests were used for the comparison of DDS score by levels of demographic and behavior variables. In addition, logistic regression was performed to evaluate the effect of DDS score on reported car crashes while adjusting for driving under substance influence.

## Results

### Study population

**Table 2:** Demographic Data (*N* = 228)

Item	Percent
Gender	
Male	47.4
Female	52.6
Ethnicity	
White	62.3
Asian	11.4
Other	17.3
Black or AA	8.0
Hispanic	
Yes	15.0
No	85.0
Education level	
High school	3.6
College/associates	94.6
Other	1.8
Driving location	
City or Urban	45.6
Suburban	44.3
Rural	10.1
Age	
Mean: 21.1 year	SD 1.88

There were 228 subjects included in the study (Table 2). Of the Boston group, 70 of 79 initial respondents completed the survey at the two-week interval and 14 respondents were additionally excluded for reporting not having driven a motor vehicle in the prior 30 days on one or both surveys. Therefore there were a total of 56 Boston respondents (25 male, 31 female). There were 90 respondents in the Eastern Region and 82 in the Western region.

Of the 228 total respondents, 120 (52.3 %) were female. Participants self-identified as White (63.3 %), Asian (11.4 %), Black/African American (8.0 %) or other (17.3 %). 34 (15.0 %) described themselves as Hispanic. Respondents said their driving was predominantly urban (45.6 %), suburban (44.3 %), or rural (10.1 %). Most (71.5 %) respondents were either in college or had completed some or all of college. Other participants were in or had completed high school (26.3 %), or described their educational status as other (2.2 %).

### Item selection: reliability

The survey was first tested in a Boston metropolitan area cohort (*N* = 56) and items were reduced based on Cronbach’s alpha and the Kappa statistic (Tables 3 and 4). Eliminated questions asked about use of voice recognition software and riding with a driver who texted, as well as use of specific anti-texting programs, all of which did not meet reliability or validity criteria. To keep the survey brief and focused, questions that were not cell-phone specific were also eliminated (i.e., drowsiness when driving, driving under the influence, seatbelt use) even though these questions were statistically reliable. There were 11 items in the final questionnaire; the Spearman correlation coefficient for test-retest reliability was excellent at 0.82 for the final survey based on the Boston data (*N* = 56) (Tables 3, 4 and 5).

**Table 3:** Test-retest reliability in the Boston Metro Area cohort (*N* = 56)

Sample items	Spearman correlation coefficient (test retest)	*p* value	Kappa
1. Think it safe to text and drive	0.67112	<.0001	0.52
2. Read text messages when driving^R^	0.726	<.0001	0.56
3. When read text messages while driving^R^	0.791	<.0001	0.67
4. Read email when driving^R^	0.683	<.0001	0.62
5. When read emails while driving^R^	0.751	<.0001	0.59
6. View maps or directions on phone while driving^R^	0.717	<.0001	0.63
7. Write text messages while driving^W^	0.529	<.0001	0.44
8. When write text while driving^W^	0.656	<.0001	0.54
9. Write emails when driving^W^	0.457	0.0004	0.32
10. When write emails while driving^W^	0.515	<.0001	0.34
11. View messages on social media sites while driving^R^	0.38	0.0041	0.33
Have been a passenger when others are texting and driving	0.655	<.0001	0.54
Drive under the influence	0.654	<.0001	0.45
Fall asleep when driving	0.376	0.0043	0.30
Use of seatbelt when driving	0.569	<.0001	0.65
Car crashes reported			0.62
Enrolled in programs to reduce texting (e.g.AT&T’s “It Can Wait”)			0.67

R indicates items in the reading subscore (Items 2–6, 11)

W indicates items in the writing subscore (Items 7–10)

**Table 4:** Distracted driving score and subscore reliability and correlation to reported crashes (*N* = 228)

	Cronbach’s Alpha (standardized)	Spearman correlation coefficient (test-retest)*	Correlation to crash rate: odds ratio per point increase	Correlation to crash rate: 95 % confidence interval	Correlation to crash rate: *p* values
DDS	0.93	0.82	1.07	1.03–1.12	*p* = 0.0005
DDS-reading	0.85	0.82	1.13	1.05–1.21	*p* = 0.001
DDS-writing	0.86	0.63	1.17	1.06–1.29	*p* = 0.0015

* Boston cohort only (N=56)

**Table 5:** Survey responses (%) (*N* = 228)

	Always	Most of the time	Some of the Time	Rarely	Never
Do you think that you can safely text and drive?	7.02	8.77	20.18	27.63	36.4
For each of the following questions, please choose the answer that best applies:					
	Every time I drive	Most of the times I drive	Some of the times I drive	Rarely	Never
In the last 30 days, have you READ text messages while driving?	2.19	13.16	27.19	28.95	28.51
In the last 30 days, have you READ email while driving?	2.63	2.19	9.65	20.61	64.91
In the last 30 days, have you viewed maps or directions on your phone while driving?	4.82	17.11	36.4	16.23	25.44
In the last 30 days, have you WRITTEN text messages while driving?	3.95	3.51	15.79	35.96	40.79
In the last 30 days, have you WRITTEN email while driving?	1.75	3.51	1.75	11.4	81.58
In the last 30 days, have you read messages or viewed information on social media apps or sites while driving?	2.19	4.39	14.91	18.42	60.09
In the last 30 days, have you driven while impaired by any substance (e.g. alcohol, marijuana)?	1.75	3.51	3.07	8.77	82.89
For each question below, please indicate the HIGHEST SPEED that you have performed the action (meaning, the column furthest to the left that is applicable) in the last 30 days					
	While driving at any speed	While driving at low speeds (under 25 mph)	While in stop-and-go traffic	While stopped at a red light	None of the above
In the last 30 days, WHEN have you READ text messages?	13.10	16.16	10.48	37.55	22.71
In the last 30 days, WHEN have you READ email?	4.80	7.02	8.33	25.44	54.82
In the last 30 days, WHEN have you WRITTEN text messages while driving?	10.09	10.09	14.91	34.21	30.70
In the last 30 days, WHEN have you WRITTEN email while driving?	3.95	4.82	5.70	18.86	66.67

The DDS-Reading or viewing subscore included six items (2–6, 11). The DDS-Writing subscore included four items that asked about specific writing activities including writing texts and emails and at what speeds (7–10). The Spearman coefficient for the DDS-Reading subscore was similar at 0.82 but lower for the DDS-Writing subscore at 0.63 (Table 5). Strong agreement was generally observed for the items included in the DDS. In addition, very good agreement was observed for most of the variables used for concurrent validity testing of the DDS including reported crashes in the last 12 months (Kappa = 0.6).

### Internal consistency

The 11-item survey with additional demographic questions was then tested in the Eastern and Western US populations. Standardized Cronbach’s alpha for the final 11-item DDS was excellent at 0.92 (*N* = 228) (Table 5). The DDS-Reading subscore standardized Cronbach’s alpha was 0.86. The DDS-Writing score standardized Cronbach’s alpha coefficient was 0.85.

### Score distribution and association with car crashes

**Fig. 1:**
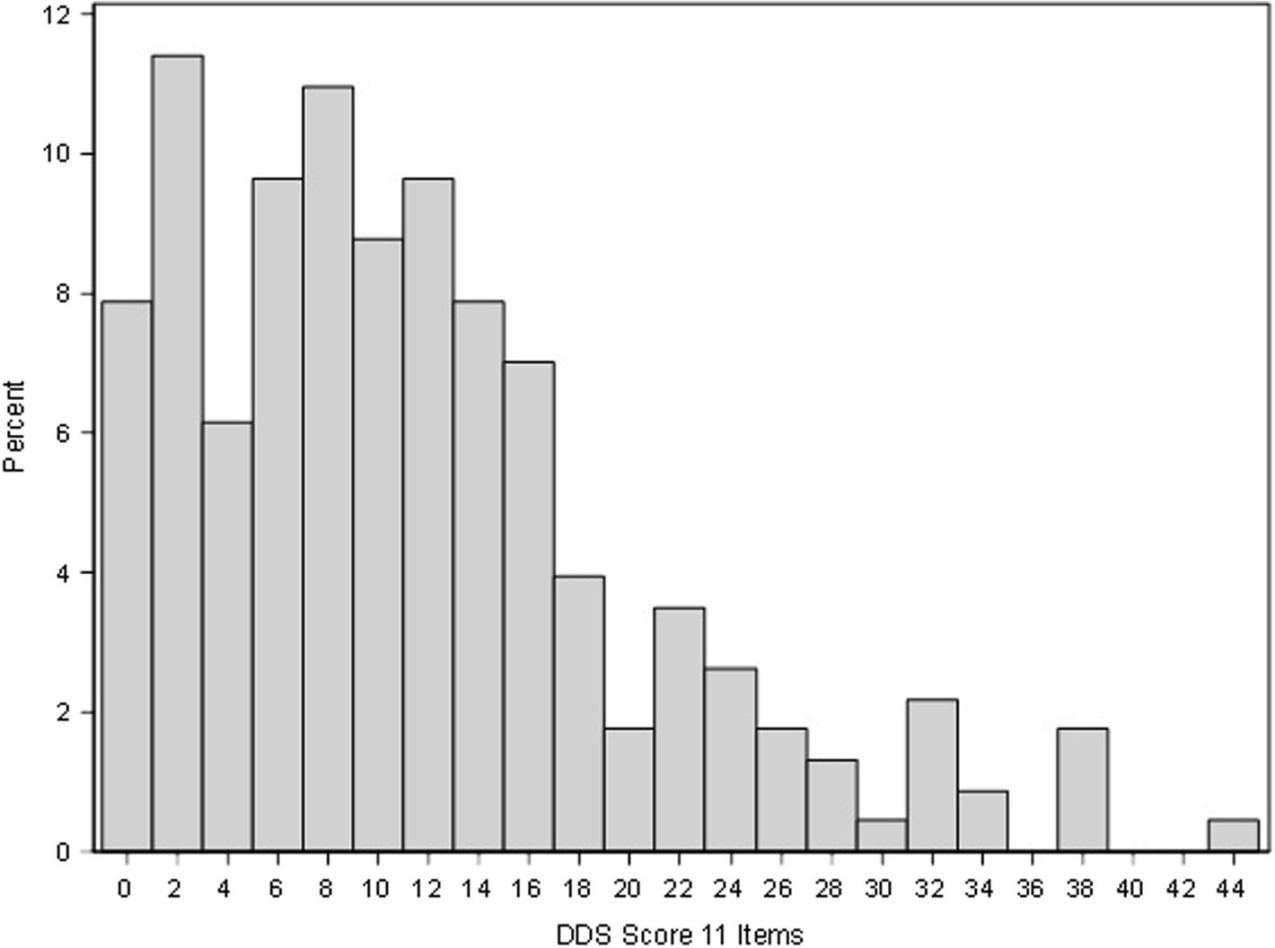
Distribution of the Distracted Driving Survey (DDS) scores. Scores reflect the final 11-item questionnaire, calculated with a range of 0 to 44 with high scores indicating more distraction

The 11-item questionnaire was then used to calculate the DDS score as described in the methods section with a higher score indicating more risk behaviors. Mean DDS score based on the entire cohort (*N* = 228) was 11.0 points with a standard deviation (SD) of 8.99 and a range of 0 to 44 points. The distribution of scores is shown in Fig. 1. There was no statistically significant difference of DDS total score by region (*p* = 0.81). The mean scores for were similar for Boston (11.2, standard deviation 7.14), Eastern United States (11.4, standard deviation 9.48), and Western United States (10.5, standard deviation 9.62).

Reading and writing scores specific subscores were also calculated and also significantly correlated with crash rate (Table 5). Mean writing score was 3.2 (SD 3.48, range 0–16), and mean viewing reading score was 6.57 (SD 5.16, range 0–24).

A higher DDS score indicating higher risk behavior was significantly associated with the self-reported car crashes (Wilcoxon rank sum test, *p* = 0.0005). Logistic regression was performed with reported car crashes as the dependent variable and DDS as the independent variable. For every one point increase of the DDS score, the odds of a self-reported car crash increased 7 % (OR 1.07, 95 % confidence interval 1.03 – 1.12, *p* = 0.0005). The odds ratio for the DDS-Writing subscore (OR 1.17) was the highest among the scores and subscores. As anticipated, DDS score was not significantly associated with either falling asleep while driving (*p* = 0.11) or driving under the influence (*p* = .09) in the Boston group (*N* = 56), and these questions were eliminated for the Eastern and Western US groups.

In order to better characterize the risk of higher DDS, the DDS-11 score was categorized into < =9, 9–15 and >15 using its median (9 points) and third quartile (15). The odds of car crash for subjects with DDS-11 > 15 is 4.7 times greater than that of subjects with DDS score < =9 (95 % CI 1.8–12.6).

### Texting and driving behavior

In this cohort of 228 18–24 year old divers (Table 5), we found that 59.2 % reported writing text messages while driving in the prior 30 days. Of the 228 drivers, most wrote text messages never or rarely, while 16 % said they write text messages some of the times they drive and 7.4 % said they write text messages most or every time they drive. When all participants were asked about the speeds at which they write text messages, 9.7 % said they write text messages while driving at any speed and an additional 24.1 % said they write text messages at low speeds or in stop and go traffic, with the remainder writing text messages only at stop lights or not writing text messages while driving at all.

Reading text messages was even more common, with 71.5 % of participants saying they read text messages while driving in the past 30 days – 29.0 % rarely, 27.2 % sometimes, 13.2 % most of the time, and 2.2 % every time they drove. Compared to writing texts, a higher percentage read text messages at any speed (12.7 %) and at low speeds (15.6 %), in stop and go traffic (10.1 %), as well as when stopped at a red light (36.3 %). Reading and writing email and browsing social media were less common. Maps were used on a phone by 74.6 % of respondents in the last 30 days.

In contrast to yes/no answers in other surveys about safety of texting and driving, this study found that only 36.4 % of respondents said it was never safe to text and drive. Drivers reported that it was safe to text and drive never (36.4 %) rarely (27.6 %), sometimes (20.2 %), most of the time (8.8 %) and always (7.0 %).” This is in contrast to yes/no answers in other surveys about texting and driving safety.

## Discussion

The purpose of this study was to create a short validated questionnaire to assess texting while driving and other cell-phone related distracted driving behaviors. The Distracted Driving Survey developed in this study proved to be valid and reliable in a population of 18–24 year old drivers, with excellent internal consistency (Cronbach’s alpha of 0.93). The DDS has excellent internal consistency defined as Cronbach’s alpha =0.9 or greater and strong test retest reliability.(Kline 1999) The Mini-DBQ, a valid measure which does not include texting or other cell-phone related distracted driving, is considered a valid measure with Cronbachs alpha of less than 0.6, substantially lower than the DDS (Martinussen et al. 2013).

The Distracted Driving Survey score was significantly correlated with self-reported crash rates in the prior 12 months with people in the highest tercile of derived scores (here, those with a score >15) more than 4.7 times as likely to have had a crash than subjects with scores in the lowest tercile of risk (here, those <9). Stepwise logistic regression demonstrated this relationship to have a ‘dose response’, with higher scores incrementally associated with higher crash rates. The odds of a reported crash increased 7 % for every increase of one point of the DDS score (OR 1.07, 95 % confidence interval 1.03 – 1.12, *p* = 0.0005). This relationship was further demonstrated to be independent of such factors as driving under the influence of alcohol or other substances, and falling asleep while driving.

The DDS confirmed prior reports of high levels of texting while driving, and further elucidated specific aspects of the behavior including to what extent people read versus write text messages and and what speeds they perform these activities. 59.2 and 71.5 % of respondents said they wrote and read text messages, respectively, while driving in the last 30 days. Respondents were most likely to do these activities while stopped, in stop-and-go traffic or at low speeds although a small percentage said they have read or written text messages while traveling at any speed. Prior studies have shown high rates of texting while driving in spite of high rates of perceived risk. In this study, Likert-scale questions further demonstrated that most respondents actually felt that texting and driving can be safe at least on rare occasions; only 36.4 % of respondents said it was always unsafe to text and drive. These data correspond more directly to the amount of texting and driving reported here including reading or writing texts while stopped or in stop and go traffic.

Texting and other cell phone use while driving is frequently targeted as a public health crisis, but many of these interventions have unclear impact. Since the advent of the Blackberry in 2003 and the first iPhone in 2007, texting and driving has been highlighted in the news and by cell phone carriers, such as with AT&T’s It Can Wait pledge, to which more than 5 million people have committed (AT&T 2014). There are multiple smartphone applications and other interventions aimed at reducing texting and driving (Verizon Wireless 2014; Lee 2007; Moreno 2013), and Ford has even created a Do Not Disturb button in select vehicles blocking all incoming calls and texts (Ford 2011). Forty-four U.S. states and the District of Columbia ban texting and driving, with Washington State passing the first ban in 2007 (Governors Safety Highway Association 2014), and there is a push for even more aggressive laws and enforcement (Catherine Chase 2014). Texting bans have been shown to be effective in some studies. Texting bans are associated with reductions in crash-related hospitalizations (Ferdinand et al. 2015). Analysis of texting behavior from the U.S. Centers for Disease Control and Prevention 2013 National Youth Risk Behavior Survey showed that text-messaging bans with primary enforcement are associated with reduced texting levels in high school drivers, whereas phone use bans were not (Qiao and Bell 2016). Other studies surveying drivers have found a mixed response of whether behavior is altered, with some drivers not altering their behavior (Mathew et al. 2014). However, the impact of many of these interventions has not yet been studied or fully understood. While driver reported surveys exist today, in general these instruments have high respondent burden and have not been designed or validated for individual measurement.

We aimed to develop a validated, reliable and brief survey for drivers to report and self-assess their level of risk and distraction to fill gaps in the literature and facilitate standardized measurement of behavior. Initial validation detailed here focused on a population of young drivers most at risk for motor vehicle crashed and deaths. Survey development was carefully undertaken here with semi-structured interviews, pilot testing and testing of young adults in a major metropolitan area as well as in the Western and Eastern United States. Validity and reliability were measured in multiple ways. While there are multiple functions associated with cell phone use that can be distracting to a driver, we focused on typing and reading or viewing activities as those have been both extensively studied and demonstrated to have significant effect sizes in the simulator literature (Caird et al. 2014).

The resulting survey is brief and easy to administer. In automated testing, the full research survey required approximately four and a half minutes to complete and completing the 11-item DDS component takes around two minutes. In actual testing, all respondents were able to complete the survey.

This survey provides self-reported data from young US drivers in a relatively small sample size of 228 drivers age 18–24. Participants voluntarily took the survey so it is possible that the type of driver who took the survey may be more attuned to the risks of texting and driving or that there may be some other selection bias. Tradeoffs were made in the comprehensiveness of the questions selected to purposefully construct a brief instrument, with intentional elimination of questions on certain functions of cell phone use and other forms of distraction. For example, this study did not quantify the driving patterns of the respondents in the prior 30 days. Respondents who had not driven in the last 30 days were excluded. Because this study aimed to validate this survey among young people age 18–24, there are college students included who may have more limited driving patterns. Further studies are needed to validate this survey among drivers of all ages. This survey did not aim to quantify the number of texts or viewing time per mile. Further studies could be done to validate this survey against quantitative measures of viewing and reading behavior, which was beyond the scope of this study. However, the high Cronbach’s alpha and other characteristics suggest that the resulting brief instrument is well suited for large population studies that seek to limit respondent burden. Further research will likely lead to refinement in the scoring algorithms used. The performance of the DDS has not yet been studied in older age groups. Strengths of the study include good ethnic representation closely aligned with US census data and an anonymous format conducive to more accurate reporting of these behaviors.

The DDS is intended to be used to assess behavior patterns and risk and to evaluate the impact of public health interventions aimed at reducing texting and other cell phone-related distracted driving behaviors. The DDS score demonstrated strong performance characteristics in this validation study. Further research is needed to evaluate the instrument in larger and more diverse populations and to evaluate its sensitivity to change following interventions. Since a DDS score can be immediately generated at the time the DDS is completed, another area of research is whether the score itself may have value as an intervention.

## Conclusion

The Distracted Driving Survey is a brief, reliable and validated measure to assess cell-phone related distraction while driving with a focus on texting and other viewing and writing activities. This survey is designed to provide additional information on frequency of common reading and viewing activities such as texting, email use, maps use, and social media viewing. The data are informative because different anti-distraction interventions target various aspects of cell phone utilization. For example, some anti-texting cell phone applications would not affect maps viewing, email viewing or writing, or social media use and therefore would not impact those behaviors. Further research is required to determine if these trends also hold true for older drivers. Higher DDS scores, indicating more distraction while driving, were associated with an increase in reported crashes in the prior 12 months in a dose–response relationship. Although this finding does not prove causality, the association is concerning and corroborates other studies demonstrating the risks of texting on crash rates on courses and simulators. This study confirmed prior reports of high rates of texting and driving in a young population, with more detailed reports of behavior on writing and reading text messages, the speeds at which these activities are performed, and respondents’ perception of risk. This measure may be used for larger studies to assess distracted driving behavior and to evaluate interventions aimed at reducing cell phone use, including texting, while driving. An improved understanding of the common cell phone functions used by young drivers should be used to inform the interventions aimed at reducing cell phone use while driving.
